# Cruel Intentions? HIV Prevalence and Criminalization During an Age of Mass Incarceration, U.S. 1999 to 2012: Erratum

**DOI:** 10.1097/01.md.0000490129.50039.31

**Published:** 2016-07-29

In the article “Cruel Intentions? HIV Prevalence and Criminalization During an Age of Mass Incarceration, U.S. 1999 to 2012”,^1^ which appeared in Volume 95, Issue 16 of *Medicine*, a reference citation in the footnote of Table 2 was given incorrectly in the original article. The article has since been corrected online.
